
JOURNAL INFORMATION
==============================
NLM Title Abbreviation: Wirtschaftsdienst
Journal ID: Wirtschaftsdienst
NLM Journal ID: 101086612

Wirtschaftsdienst (Hamburg, Germany : 1949)

ISSN: 0043-6275

ARTICLE INFORMATION
==============================
PMCID: PMC7368603
PMID: 32834161
DOI: 10.1007/s10273-020-2684-5
Article ID: 2684
Article version: 1
Subjects: Kurz Kommentiert

V-Erholung unwahrscheinlich

Supply chains: A V-shaped recovery is unlikely

Marin Dalia Dalia.Marin@lrz.uni-muenchen.de

grid.6936.a 0000000123222966 TUM School of Management, Technische Universität München, Arcisstrasse 21, 80333 München, Deutschland
Electronic publication date: 2020 Jul 18
Print publication date: 2020
Volume: 100
Issue: 7
First page: 476
Last page: 476
Copyright: © Der/die Autor(en) 2020
License: Open Access Dieser Artikel wird unter der Creative Commons Namensnennung 4.0 International Lizenz veröffentlicht, welche die Nutzung, Vervielfältigung, Bearbeitung, Verbreitung und Wiedergabe in jeglichem Medium und Format erlaubt, sofern Sie den/die ursprünglichen Autor(en) und die Quelle ordnungsgemäß nennen, einen Link zur Creative Commons Lizenz beifügen und angeben, ob Änderungen vorgenommen wurden.
Die in diesem Artikel enthaltenen Bilder und sonstiges Drittmaterial unterliegen ebenfalls der genannten Creative Commons Lizenz, sofern sich aus der Abbildungslegende nichts anderes ergibt. Sofern das betreffende Material nicht unter der genannten Creative Commons Lizenz steht und die betreffende Handlung nicht nach gesetzlichen Vorschriften erlaubt ist, ist für die oben aufgeführten Weiterverwendungen des Materials die Einwilligung des jeweiligen Rechteinhabers einzuholen.
Weitere Details zur Lizenz entnehmen Sie bitte der Lizenzinformation auf http://creativecommons.org/licenses/by/4.0/deed.de.
License URL: https://creativecommons.org/licenses/by/4.0/

issue-copyright-statement: © The Author(s) 2020

==============================
DOI: 10.1007/s10273-020-2684-5

Lieferketten

### V-Erholung unwahrscheinlich

Lange waren die globalen Lieferketten die stillen Motoren der wirtschaftlichen Globalisierung. Von 1990 bis 2008 trieben sie die rapide Ausweitung des Handels an; 60 % bis 70 % des Handelswachstums entfielen auf sie. Inzwischen jedoch sind sie zum Stillstand gekommen. Der auf den globalen Lieferketten lastende Druck spiegelt die Hinwendung vieler Regierungen zu einer protektionistischen Politik wider. Und jetzt hat die COVID-19-Pandemie eine durch einen Angebotsschock bedingte Rezession verursacht. Die damit verbundene Unsicherheit könnte die Expansion der globalen Wertschöpfungsketten um mindestens 35 % verlangsamen. Tatsächlich wächst der Welthandel inzwischen nicht mehr schneller als das globale Bruttoinlandsprodukt (BIP). Setzt sich dies fort, werden viele Unternehmen ihre Fertigung aus Asien und anderswo zurückholen.

Die schrumpfende Produktion wird weltweit eine Rezession – und Erholung – hervorrufen, wie wir sie noch nie erlebt haben. In ihren Prognosen für 2021 gehen der Internationale Währungsfonds, die OECD und andere internationale Organisationen von einer V-förmigen Erholung aus. Doch wurde dieses Narrativ vermutlich durch die rasche Erholung der globalen Wertschöpfungsketten nach der Großen Rezession von 2008 bis 2010 beeinflusst, einem Abschwung, der seinen Ursprung im Finanzsystem und nicht weltweit in der Realwirtschaft hatte. Angesichts der Bedeutung zerbrochener Lieferbeziehungen im derzeitigen Abschwung dürfte diese Rezession einzigartig ausfallen. Unternehmen sind noch auf andere Weisen anfällig. So können von einem Lockdown betroffene Lieferanten ihren Kunden erhebliche Produktionsverluste zufügen, wenn die von ihnen produzierten Vorprodukte kundenspezifischer Art sind und ein hohes Maß an Forschung und Entwicklung und an geistigem Eigentum auf sich vereinen. Eine Umstellung auf einen anderen Lieferanten ist dann kostspielig und langwierig.

Es überrascht nicht, dass pandemiebedingte Störungen einzigartig sind. Bei der Untersuchung bedeutender Naturkatastrophen in den USA haben Jean-Noël Barrot und Julien Sauvagnat vom MIT festgestellt, dass von einer Überflutung, einem Erdbeben oder einem ähnlichen Ereignis betroffene Lieferanten ihren Kunden große Produktionsverluste zufügen. Tatsächlich erlitt, wenn ein Lieferant von einer derartigen Katastrophe betroffen war, das Umsatzwachstum der Unternehmen einen durchschnittlichen Rückgang von 2 bis 3 Prozentpunkten. Die Auswirkungen griffen auf weitere Zulieferer über und vergrößerten die ursprüngliche Erschütterung.

Es ist zudem wahrscheinlich, dass diese Rezession ein niedrigeres BIP-Trendwachstum hervorbringen wird. Die globalen Lieferketten waren in den 1990er Jahren und während des größten Teils des folgenden Jahrzehnts ein wichtiger Antriebsfaktor für das Produktivitätswachstum in vielen Ländern. Die Expansion der Lieferketten nach Osteuropa nach dem Mauerfall trug nicht nur zur Erholung Deutschlands bei, sondern löste auch in Tschechien, Ungarn, Polen, der Slowakei und anderen Ländern der Region ein starkes Wachstum aus. Wenn schon die Verlangsamung des Wachstums der globalen Wertschöpfungsketten seit 2011 zu blutleerem Produktivitätswachstum in den entwickelten Ländern beigetragen hat, lässt eine noch stärkere Verlangsamung oder gar Kontraktion nichts Gutes erwarten.

Unter diesen Umständen besteht die einzige Option für die Politik darin, das Wachstum in bestimmten Sektoren anzukurbeln. In Deutschland hat die Autoindustrie auf eine Abwrackprämie ähnlich wie 2009 gedrängt. Die Bundesregierung jedoch hat sich gegen eine derartige Maßnahme entschieden. Dies ist überdenkenswert. Unter normalen Umständen würde man eine Konjunkturpolitik, die bestimmte Sektoren bevorzugt, ablehnen. Neue makroökonomische Modelle legen jedoch nahe, dass sektorenspezifische Konjunkturmaßnahmen die größten fiskalischen Impulse erzeugen könnten. Eine Volkswirtschaft, in der 50 % der Wirtschaft pandemiebedingt komplett heruntergefahren wurde, ist nicht dieselbe wie eine, in der die gesamte Wirtschaftsaktivität in einer Depression um 50 % einbricht. In einer Pandemie kommt es auf die Beziehung eines Sektors zum Rest der Volkswirtschaft an.

Der beste Weg, um die fiskalischen Konjunkturimpulse zu maximieren, besteht in der Ermittlung nicht-substitutiver Sektoren. Autos haben eine Komplementärbeziehung zum Rest der Volkswirtschaft. Je mehr Autos konsumiert werden, desto größer ist die Nachfrage nach Autovorleistungen. Die Branche importiert lediglich 29 % ihrer Vorprodukte. Daher sind Programme zur Ankurbelung von Autokäufen gegenüber Restaurantgutscheine zu bevorzugen. In diesen schwierigen Zeiten soll sich die Regierung auf den Wiederaufbau konzentrieren und Umwelt und technologische Auflagen hintanstellen. Es besteht sonst die Gefahr, dass keines dieser Ziele erreicht wird. Umwelt- und Strukturwandel haben eine herausragende Bedeutung, aber zuerst muss der Wiederaufbau gelingen.

Dalia Marin
Technische Universität München
dalia.marin@tum.de

© Der/die Autor(en) 2020. Open Access: Dieser Artikel wird unter der Creative Commons Namensnennung 4.0 International Lizenz (https://creativecommons.org/licenses/by/4.0/deed.de) veröffentlicht.

Open Access wird durch die ZBW – Leibniz-Informationszentrum Wirtschaft gefördert.
